# From the Editor’s Desk

**Published:** 2016

**Authors:** Commerford PJ

Acquired von Willebrand syndrome (AVWS) is a rare clinical condition characterised by prolonged bleeding time and decreased levels of factor VIII and von Willebrand factor. It has been reported to occur in patients with severe aortic stenosis and other cardiac conditions associated with high shear stress, such as para-valvar leaks after prosthetic valve replacement surgery. First reported decades ago, the mechanism was initially unclear but it has now become known. In this issue, Binnetoğlu and colleagues (page 222) report on a prospective series of children with aortic and pulmonary stenosis, and describe the frequency of occurrence and underlying pathophysiology.

There is general agreement on the considerable variation in the prevalence of various cardiac diseases among the different ethnic groups in Africa and there may also be differences in the manner in which the diseases express themselves. There is less clarity about the contributions of intrinsic ‘genetic’ differences to that variability compared to acquired or environmental factors. Sirtuin 1 (SIRT1) has been identified as a candidate molecule affecting the epigenetic mechanisms of cardiovascular disease (CVD). Previous studies have shown that some SIRT1 singlenucleotide polymorphisms (SNPs) are associated with body mass index, diabetes, blood pressure, cholesterol metabolism and coronary artery calcification. An investigation conducted by Ramkaran and co-workers (page 213) in young South African Indians with coronary disease concluded that SNP variant alleles occurred more frequently in South African Indians than in black South Africans. The study is not large enough to definitively assess whether these variants may serve as risk factors that contribute to Indians developing early-onset CVD but the results are intriguing and warrant further investigation.

Technological advances in many aspects of the management of CVD continue to amaze those of us who have been privileged to witness their evolution and development. Left ventricular assist devices (LVADs) and two- and three-dimensional echocardiography were the stuff of dreams when many of us were training but now are part of routine care in some parts of the world. Demirozu and co-workers (page 208) elegantly demonstrate how the use of advanced imaging techniques of echocardiography can be used to fine-tune the functioning of LVADs.

Intravascular stenting was equally unthinkable only a few decades ago but has now revolutionised much of the management of CVD. The way in which a new generation of biodegradable stents promises to advance this area even further is described by Tiryakioglu et al. (page 238).

Cardiologists and physicians trained in an era prior to the introduction of the technological advances mentioned above will be pleased to see that the old stalwart, electrocardiography (ECG) continues to be widely used. Some consider it does not receive the recognition it deserves as a cheap, non-invasive adjunct to the clinical examination. A cross-sectional study carried out on adults in Nigeria examined the ECGs of 100 HIV-infected patients on highly active anti-retroviral therapy (HAART), 100 HIV-infected HAART-naïve patients and 100 HIV-negative controls (Njoko et al., page 252). The clinical relevance of these findings and that of similar findings reported by others and discussed by the authors, remains unclear and requires long-term follow up studies accompanied by imaging, either by echocardiography or perhaps more helpfully, by cardiac magnetic resonance imaging.

